# Multidrug-Resistant Enterococcal Infection in Surgical Patients, What Surgeons Need to Know

**DOI:** 10.3390/microorganisms11020238

**Published:** 2023-01-17

**Authors:** Soroush Farsi, Ibrahim Salama, Edgar Escalante-Alderete, Jorge Cervantes

**Affiliations:** Paul L. Foster School of Medicine, Texas Tech University Health Sciences Center El Paso, El Paso, TX 79905, USA

**Keywords:** *Enterococcus*, surgical site infection, multidrug resistance

## Abstract

Enterococci are organisms that can be found in the normal intestinal and skin microbiota and show remarkable ability to acquire antibiotic resistance. This is an enormous challenge for surgeons when faced with surgical site infections caused by multidrug-resistant (MDR) Enterococci. Due to an increase in the prevalence of MDR *Enterococcus* within the last few decades, there has been a major decrease in therapeutic options, because the majority of *E. faecium* isolates are now resistant to ampicillin and vancomycin and exhibit high-level resistance to aminoglycosides, traditionally three of the most useful anti-enterococcal antibiotics. There is limited data regarding the magnitude and pattern of multidrug resistance among the enterococcal genus causing surgical site infections in hospitalized patients. The scope of the review is to summarize the most recent findings in the emergence of postoperative MDR Enterococci and discuss recent mechanisms of resistance and the best treatment options available.

## 1. Introduction

Surgical site infection (SSI) is defined as the presence of pathogenic microorganisms, which have developed in an incision site either within the skin and subcutaneous fat and musculofascial layers or in an organ or cavity [1]. SSIs account for 15% of all nosocomial infections and, among surgical patients, represent the most common nosocomial infection [2]. SSIs are associated with significant clinical and economical burdens, including longer hospital stays and an increased risk of readmission. SSIs are a major cause of postoperative morbidity and death in the U.S. health system. Not only do they affect the rehabilitation process, but they also increase hospital stay length and cost [3,4,5], drastically escalating expenses, causing higher rates of hospital readmission [6], and jeopardizing health outcomes [2]. The rate of hospital-acquired SSIs is markedly higher in developing countries, partially due to the performance of surgical procedures without proper postoperative management [7].

Management of hospital-acquired SSIs is further complicated due to the increasing appearance of multidrug-resistant organisms such as vancomycin-resistant enterococci (VRE), multidrug-resistant (MDR) enterococcal species, methicillin-resistant *Staphylococcus aureus* (MRSA), and methicillin-resistant coagulase-negative staphylococci (MRCNS) [7]. SSIs caused by Gram-positive infections are very common in the U.S., and the prevalence of nosocomial bloodstream infections caused by multidrug-resistant, Gram-positive bacteria has been shown to be increasing [8].

We herein present a comprehensive and up-to-date revision of the literature on surgical infection caused by enterococci, with the goal of providing information useful for those working in the surgical field.

## 2. The Genus *Enterococcus*

The history of *Enterococcus* started in 1899 with the description by Thiercelin and Jouhaud of a new Gram-positive diplococcus, which was later included in the new genus Enterococcus with the type species *Enterococcus proteiformis*. In 1906, however, Andrewes and Harder renamed Thiercelin’s ‘Enterocoque’ as *Streptococcus faecalis* based on its ability to form short or long chains. Because of these early links, the history of enterococci cannot be considered separately from that of the genus *Streptococcus*. In the 1930s, enterococci were classified as group D streptococci, along with *Streptococcus bovis*. In the 1980s, the genus *Enterococcus* was separated from the genus *Streptococcus* and received its own taxonomy based on genetic differences in the group D streptococci [9].

The source of nosocomial enterococci infection has been thought to be endogenous, particularly in operations involving the urinary tract, oral cavity, and the gastrointestinal tract. Despite the endogenous nature of the host’s microbiota, the hospital environment and the severity of patient illness may render endogenous commensals more invasive. The large increase in gentamicin-resistant enterococcal surgical site infections has been reported to be due to transmission by direct contact with medical personnel [10].

Since the early 2000s, *Enterococcus* colonization has been considered endemic in most U.S. intensive care units (ICUs), with a prevalence rate of 28%. Vancomycin-resistant *E. faecium* makes up most of the enterococcal infections in U.S. clinical settings [11]. In Europe, hospital-acquired enterococcal infections have also been on the rise since the early 2000s. Based on the European Antimicrobial Resistance Surveillance System annual epidemiological report for 2021, *E. faecalis* had a prevalence of 8.8%, and *E. faecium* had a prevalence of 6.2%, with four countries reporting a prevalence higher than 28% (Greece, 66.3%; Romania, 48.3%; Italy, 29.5%; and Bulgaria, 28.1%) [12]. Nosocomial VRE first started to appear in Chinese surgical ICUSs and emergency ICUs in 2003. Unlike in the U.S. and Europe, where the vanA type of VRE is more commonly seen, vanB phenotype–vanA genotype VRE strains are most commonly found in the East Asian region, i.e., in Japan, Korea, and Taiwan [13].

## 3. Cost of MDR *Enterococcus* Infections for the U.S. Healthcare System

Antimicrobial resistance is a serious concern and a growing public health threat. Antibiotic-resistant pathogens are associated with greater mortality, morbidity, and costs compared to infections caused by susceptible organisms. The national costs of antimicrobial resistance in the U.S. have been estimated to be up to USD 30 billion annually. A study performed in 2002 found that antibiotic-resistant enterococci culture positivity was associated with a 2-fold increased odds of mortality, a 2.7-fold increased odds of a major surgical procedure, a 3.5-fold increased odds of admission to the ICU, a 1.7-fold increase in length of hospital stay, a 1.4-fold increase in cost of hospitalization, and a 2-fold increased odds of discharge to a long-term care facility [14]. A different situation, however, may be seen in Europe, as a recent analysis from Germany on hospital mortality and enterococci-infection-attributed hospital stays found that these variables may be influenced by *Enterococcus* species and underlying diseases rather than by vancomycin resistance [15]. Therefore, future studies should consider adjusting for Enterococcus species in addition to vancomycin resistance in order to provide a conservative estimate for the burden of VRE infections.

Patients who survive antibiotic-resistant *Enterococcus* infection tend to have more complications later on, with a higher chance of them needing long-term care treatment after discharge. Having an antibiotic-resistant enterococci infection has been associated with a higher rate of being discharged to long-term care [14]. A similar scenario has been described in Europe, where VRE infections significantly increase hospital costs compared with VSE infections [16]. 

## 4. Identifying Clinically Significant *Enterococcus* Species

Enterococci are ovoid-shaped, Gram-positive bacteria that can be found singly, in pairs, or in chains. They are facultative, anaerobic, and do not form spores. Most members of the family are oxidase, urease, and catalase negative while being tolerant to salt levels up to 6.5% and bile up to 40%. While they can grow in a wide temperature range, from 10 to 45 °C, optimal growth is obtained in the 35–37 °C window. They have a wide variability of hemolytic activity when grown on blood agar and differ between species. Despite the mentioned characteristics, selective agar media cannot differentiate between species of enterococci.

Since enterococci are common players in nosocomial infections, quickly identifying them in the hospital setting is an invaluable tool for healthcare workers. *E. faecalis* and *E. faecium* are most notable as they are widely regarded as the most common species from the *Enterococcus* genus causing disease. Despite this, new species are being discovered. While the majority of enterococcal species do not cause disease, and do not even inhabit humans, there is a growing number of *Enterococcus* species, such as *E. canintestini, E. durans, E. gilvus, E. pallens*, and *E. sanguinicola*, that infect humans [17]. As mentioned in the sections above, enterococci have become an increasing dilemma in the medical field due to their growing propensity for antibiotic resistance. Different *Enterococcus* species have varying intrinsic and acquired susceptibility capabilities. Species identification along with extensive knowledge of susceptibility profiles can influence treatment plans and improve therapeutic outcomes.

As technology has improved in the clinical setting, new molecular techniques which allow for more specific and accurate species identification have been devised. These newer molecular techniques, however, are more costly and are not available in many clinical settings. Some examples are matrix-assisted laser desorption ionization–time of flight mass spectrometry (MALDI-TOF MS), nucleic acid amplification tests (NAATs), peptide nucleic acid fluorescent in situ hybridization (PNA-FISH), and multilocus sequence typing (MLST) [18]. MALDI-TOF MS brings increased specificity to the table, being able to identify 36 strains from different species of *Enterococcus*, as well as almost 94% of isolates to their specific species, thus, allowing for differentiation between very similarly related species [18,19]. It has also been reported that MALDI-TOF MS can be used to detect the presence of different van genes, which will allow for in-depth antibiotic susceptibility profiling in the near future [18]. NAATs are a well-known technique that utilizes polymerase chain reaction (PCR) amplification. It has been shown to be able to accurately differentiate between *Enterococcus* species, as well as between subspecies, albeit at a less accurate level. PNA-FISH has also been shown to be able to discriminate between different *Enterococcus* species by targeting species-specific ribosomal ribonucleic acid (rRNA). Lastly, MLST’s ability to efficiently identify different strains has made it a selected epidemiological tool to study outbreaks [18]. 

## 5. The Role of *Enterococcus* in Polymicrobial Infections

Many SSIs are polymicrobial infections, wherein many bacteria coexist on or within the host tissue. Polymicrobial environments within a tissue or on a medical device help the bacteria to become more tolerant to antibiotic treatment or environmental stresses. Biofilm structure formation is a defense mechanism utilized by some species of bacteria. Many different microorganisms can live within biofilms made by other bacteria and use it as a hub for horizontal gene transfer to become even more resistant to stressors. 

Enterococci are significantly involved in polymicrobial infections, especially in those with immunocompromised immune systems or in those with underlying health conditions. Since the 1980s, polymicrobial infections of the catheterized urinary tract, wounds, diabetic soft tissues, bloodstream, and intra-abdominal and pelvic sites have been reported to be Enterococci associated [20]. Bacterial species that are commonly seen infecting along with *Enterococcus* in polymicrobial infections include *Staphylococcus aureus, Escherichia coli, Pseudomonas aeruginosa, Klebsiella spp.,* and *Proteus spp*. Studies on the *Caenorhabditis elegans* model reported increased lethality of worms co-infected with *E. faecalis* and *E. coli*, associating enhanced mortality with polymicrobial infections [20].

## 6. Surgical Site Infection by *Enterococcus*

Surgery is the main cause of most nosocomial infections in the world. Postoperative SSIs are a common healthcare problem among surgically treated patients. The development of an SSI is due to the microbial contamination of the surgical wound, either endogenously or, less frequently, from exogenous sources [21]. SSIs can be superficial infections involving the skin or involve the underlying tissue and organs, which leads to more serious outcomes. They often involve multiple pathogens living together. In general, when the microbial concentration is higher than 104 microorganisms per gram of tissue, there is a potentially high risk of developing an infected wound [22].

Enterococci were previously considered commensal organisms of little clinical importance but have emerged as serious nosocomial pathogens responsible for endocarditis and infections of the urinary tract, bloodstream, meninges, wounds, and biliary tract, among others [23]. Two main, dominant pathogenic species within the *Enterococcus* genus are *Enterococcus faecalis*, which accounts for about 90% of infections, and *Enterococcus faecium*, which accounts for 7–8%, followed by several less common species (*E. durans, E. avium, E. raffinosus, E. gallinarum, E. casselflavus, E. flavescens*) [24].

Although many studies have discussed enterococcal infections, their pathogenicity, and associated risk factors in hospital settings, few studies have focused on enterococcal infection in surgical sites in postoperative patients (Table 1). Subjects with enterococcal SSIs have a higher incidence of multiple infections, and the majority develop at least one polymicrobial infection at the surgical site [3,4,25]. In addition, postoperative enterococcal infections have been strongly associated with prior antibiotic exposure, such as exposure to cephalosporin and ampicillin, mostly administered as prophylaxis [25,26].

## 7. Mechanisms of Enterococci Drug Resistance

MDR enterococci are important nosocomial pathogens. Shaped by the selective pressures of their competitive environment, these bacteria have evolved a diverse array of responses and genetic plasticity, allowing them to thrive in the modern healthcare environment. Enterococci are highly resilient and can survive various difficult conditions such as common antiseptics and disinfectants, promoting their widespread persistence on ordinary, inanimate hospital items [27]. They are found on the hands of healthcare workers, accounting for their easy transmission [28]. There is virtually no antibiotic group to which enterococci have not developed resistance [29,30,31] (Table 2).

When exploring the topic of resistance, the idea of intrinsic versus acquired resistance must be considered. Intrinsic resistance is described as a bacterial species’ natural resistance to either a specific antimicrobial or a whole family of antibiotics. Acquired resistance, on the other hand, is due to changes or transfer of genetic material in bacteria [27]. As a result, only the initial bacteria and its progeny have acquired resistance. The most notable form of DNA transfer between bacteria is conjugation, i.e., the horizontal gene transfer from a donor to recipient bacteria while they are in direct contact [32]. The two species that cause the majority of enterococcal infections, *E. faecalis* and *E. faecium,* both demonstrate intrinsic resistance to common antibiotics [18]. Species such as *E. faecium* bear a malleable genome which allows mobilization of genetic elements, providing adaptability of this organism in the hospital. The enterococcal pangenome is large and reflects the highly plastic nature of their genomes and particular niche adaptations [18].

One of the most prominent examples of antibiotic resistance in enterococci is their ability to evade β-lactam antibiotics. This group of antibiotics halts peptidoglycan synthesis by irreversibly reacting with penicillin-binding proteins (PBPs). There are two classes of PBPs, class A and class B. Class A PBPs have dual transglycosylase and transpeptidase activity, while class B only possesses transpeptidase activity. Enterococci species possess intrinsic β-lactam resistance due to a gene called pbp5, which encodes for a class B PBP, which has an exceptionally low binding affinity to many β-lactams, most notably cephalosporins and ampicillin [33]. *E. faecalis* has been shown to be up to 100 times more resistant to β-lactams than streptococci. In addition to this, when compared to *E. faecalis, E. faecium* has been noted to be up to 16 times less susceptible to β-lactams [23]. A second way by which *Enterococcus* is shown to resist ampicillin is through its acquisition of β-lactamase activity. β-lactamases work by cleaving the β-lactam ring, thus, leaving the antibiotic non-functional. Because this is a form of acquired resistance, not all species of enterococci have β-lactamase activity, but it has been noted in *E. faecalis* and *E. faecium* [34]. Although the acquisition of this resistance is not fully understood, it is theorized that it originated through a process of conjugation with a species of *Staphylococcus*. This resulted in the obtaining of an operon that contains three genes, including blaZ, which encodes for a β-lactamase [35].

In addition to through pbp5, enterococci have shown intrinsic resistance to cephalosporins through two-component regulatory systems (TCS) [36]. TCSs are a mechanism that bacteria use to sense environmental stimuli and respond accordingly to them. The mechanism involves the activity of a histidine kinase, which acts as a sensor to specific stimuli, and a response regulator, which, upon activation, has a downstream effect on gene expression [37]. Cephalosporin resistance in enterococci has been attributed to the TCS CroRS as the histidine kinase and CroR as the response regulator [38]. Another TCS is the serene/threonine kinase IreK [29].

Unlike antibiotic groups that target cell wall formation, aminoglycosides target the A-site on of the 16S rRNA of the 30S ribosomal subunit found in bacteria. This irreversibly inhibits formation of the bacterial initiation complex during protein synthesis [39]. Enterococci display tolerance to aminoglycosides in both an acquired and intrinsic fashion. In order for aminoglycosides to function, they must first be absorbed into the bacterial intracellular space. Enterococci have a naturally occurring poor uptake of aminoglycosides; thus, higher levels of the antibiotic are needed for sufficient concentrations intracellularly. This explains the existing bactericidal synergistic effect of a cell-wall-acting antimicrobial (such as a β-lactam) and an aminoglycoside, which has become the standard for treatment of enterococcal infections. When inside the cell, enterococcal enzymes covalently alter the aminoglycoside, thus, reducing its affinity to the A-site. This is an example of intrinsic resistance. One example of these enzymes is the 6’-acetyltransferase found in *E. faecium* known as AAC(6’)-Ii [40]. Another example is the enzyme APH(3’)-IIIa. In addition to neutralizing the antibiotic, *E. faecium* also has an acquired resistance to aminoglycosides through an rRNA methyltransferase called EfmM, which works by altering the 16S rRNA through methylation of the cytidine at position 1404. Only two antibiotics are not affected by the above methods of resistance, gentamycin and vancomycin. These two antibiotics are commonly used in concurrence with the likes of β-lactams or glycopeptides. As enterococci evolve, even gentamycin and vancomycin resistance is becoming more prevalent [40].

## 8. Vancomycin-Resistant Enterococci (VRE)

Another notable family of antibiotics that *Enterococcus* displays resistance to is the glycopeptides. Vancomycin and teicoplanin are the most prominent members of this family. In particular, vancomycin-resistant *Enterococcus* (VRE) has become a challenge to manage in the hospital setting [41].

Similar to β-lactams, they interrupt peptidoglycan production, thus, inhibiting bacterial cell wall construction. Unlike β-lactams, which target PBPs, glycopeptides target their pentapeptide precursors. This occurs when the precursors are being translocated from the cytoplasm to the surface of the cell. The precursors end with a D-Ala-D-Ala terminal that is transglycosylated, forming numerous cross-links and strengthening the peptidoglycan molecule [42].

Enterococci have developed resistance to glycopeptides in a two-step fashion. They have formed pentapeptide precursors with D-Ala-D-Lac or D-Ala-D-Ser end terminals, which glycopeptides have a much lower affinity for (~1000 fold) [43]. This change in pentapeptide precursor end terminal is due to vancomycin-resistant gene clusters which promote their phenotypic variation. There are currently nine variants: vanA, vanB, vanC, vanD, vanE, vanG, vanL, vanM, and vanN. All gene cluster variations were obtained through acquired resistance with the exception of vanC, which is an example of intrinsic resistance. VanA is the most common gene cluster and is most notably found in *E. faecium* [44].

VRE *faecalis* and VRE *faecium* were first discovered in 1988 in England and have been spreading rapidly around the world since [45]. VRE were non-existent in hospital settings in the U.S. before 1990, but, currently, 87% of *E. faecium* strains from nosocomial infections and 14% of *E. faecalis* are vancomycin resistant [46]. Not all species of enterococci show the same level of resistance to vancomycin. *E. faecium* strains exhibit a higher level of vancomycin resistance, while *E. faecalis* strains have a lower rate of becoming vancomycin resistant [24]. 

Colonization of VRE most commonly occurs in the skin and oropharyngeal and gastrointestinal tract [47]. Eradication of VRE on contaminated objects is relatively difficult because they can resist desiccation and extreme temperatures [48]. Infections with VRE often occur in patients in intensive care units, immunosuppressed hosts, particularly liver and other solid organ recipients, and patients with post-chemotherapy neutropenia, which magnifies the importance of effective and multidisciplinary antimicrobial treatment [24,49]. Although VRE infections are associated with a prolonged hospital stay, they do not increase the mortality associated with enterococcal bacteremia [50]. 

## 9. Treatment for Enterococcal Infections

Among the antibiotics that have lost their efficacy against enterococcal organisms are β-lactams, cephalosporins, aminoglycosides, quinolones, macrolides, and glycoproteins. Even more concerning, it has been noted recently that these organisms have acquired resistance to newer-generation antibiotics, including daptomycin and linezolid [51]. For this reason, it has become vital to consciously analyze the risks and benefits to each antibiotic choice due to the side effects associated with these medications without exhausting the few alternative treatments present nowadays.

Considering different cues, such as type of infection, source of infection (community acquired vs. nosocomial), and demographic information, such as age, gender, and their medical record, reason for hospitalization, and hospitalization time, is a good initial step in order to target treatment for patients. Patients receiving immunosuppressive treatments, such as treatment with glucocorticoids and anti-TNF agents, and possessing factors such as previous use of penicillins and cephalosporins, are more susceptible to having ampicillin-resistant enterococcus (ARE) [29,52]. On the other hand, patients hospitalized for fewer than five days and those who have undergone surgery are more likely to have acquired antibiotic-sensitive *Enterococcus* (ASE) [53].

Monotherapy with aminopenicillins is imperfect. The use of synergistic antibiotics that act in conjunction with ampicillin has been studied in depth in order to help combat enterococcal infections. Aminoglycosides, specifically, gentamicin and streptomycin, often benefit from the ability of ampicillin to break the cell wall of enterococcus in order to increase their capacity to concentrate inside the bacterium cell and cause damage to the 30S subunit [54]. Although this combination of antibiotics seems to work well for the most part, *Enterococcus* has found ways to evade the action of gentamicin and streptomycin, specifically through the action of aminoglycoside-modifying enzymes and ribosomal mutations. The use of cephalosporins, which act along with ampicillin to saturate the cell wall membrane of *E. faecalis,* has proven to be as effective as the use of gentamicin or streptomycin [55]. In addition, cephalosporins have also been proved to be less nephrotoxic and should be considered the first line of treatment for patients with kidney pathologies.

The use of vancomycin is generally reserved for cases where there is an allergy to ampicillin. It is important to consider the association between the use of vancomycin and increased mortality in patients on β-lactam therapy when there is no evidence of resistance to ampicillin [56]. *E. faecalis* is more susceptible to both ampicillin and vancomycin when compared to *E. faecium* [24]. 

Vancomycin resistance becomes a challenge when treating *E. faecium* infections given the few available treatment options. A limited number of antibiotics have been licensed by the Food and Drug Administration (FDA) and other agencies [57]: quinupristin/dalfopristin (Q/D) in 1999, linezolid in 2000, and daptomycin in 2003. This last antibiotic is only licensed for treatment of skin and soft tissue infections, although it has also been used for VRE bacteremia. In cases of enterococcal infections with ampicillin resistance (i.e., MIC > 64 μg/mL), the first line of treatment should be Q/D, daptomycin, or linezolid.

Q/D, a combination of streptogramin B and A, functions by inhibition of protein synthesis in the early and late phases of protein synthesis [24]. Q/D MIC against *Enterococcus* is generally registered in the 1 to 2 μg/mL range, and Q/D-resistant *Enterococcus* has rarely been reported. However, studies have shown that VRE are only susceptible at concentrations of 8 μg/mL or greater due to the presence of a dalfopristin inhibitor MLS_B_ phenotype [31]. Furthermore, *E. faealis* has intrinsic resistance to Q/D due to the presence of the *lsa* gene, an ATP binding protein [55]. Although Q/D is atypical in enterococcal treatment for VRE infections given its significant side effects, such as phlebitis, myalgias, and arthralgias, studies have shown that, when used in combination with other antibiotics, such as rifampin and doxycycline, it can be efficacious.

Additional options for the treatment of VRE are daptomycin and linezolid. Both antibiotics can be used in combination with ampicillin, tygercycline, doxycycline and rifampin, and fluoroquinolones. Daptomycin is recommended in doses of 8–12 mg/kg. In addition, combining daptomycin with streptomycin or gentamicin is highly encouraged when there is evidence of enterococcus susceptibility to aminoglycosides. A systematic review demonstrated worse outcomes, including infection-related mortality and VRE relapse, for patients treated with daptomycin compared to those treated with linezolid [58]. More interestingly, they also demonstrated that there was no difference in cure rate in patients treated with either antibiotic. In addition, daptomycin has also been implicated in the surge of daptomycin, linezolid, and VRE resistance (DLVRE), especially in patients with immunosuppression or those who have undergone recent, invasive procedures [59].

The selection of appropriate antibiotic therapy prior to the first incision on the operating room should be guided by the type of surgery and the risk factors associated with the patient. It is generally recommended that the antibiotic to be used prophylactically should be administered at least 60 min prior to the surgical procedure. Vancomycin or fluoroquinolones should be administered at least 120 min before starting the procedure. Readministration of antibiotics should be considered if their half-lives are short, as in the case of cefazolin and cefoxitin, and whenever the duration of the procedure is prolonged. Administration of a second dose should also be considered in cases where there is excessive bleeding, or if there are other factors that might shorten duration of the antibiotic effect, such as patients having renal insufficiency. In the obese patient, it is important to approach using a weight-based dose as pharmacokinetics are altered in these situations [60]. In general, prophylaxis with cefazolin or cefazolin (2–3 g), plus metronidazole (1 g) or cefoxitin (2 g), can be used in situations involving risk of enterococcus exposure during surgery or clean-contaminated surgeries. In addition, the use of piracillin–tazobactam (3.375 g) for liver transplants should be considered. Moreover, it is important to remember that strategic use of antibiotics is necessary in order to stop the increasing number of complicated surgical site infections caused by VRE. As we know, treatment options for infections with VRE are scarce (Q/D, daptomycin, and linezolid). Linezolid is associated with myelosuppression. A retrospective study assessed the outcomes of prophylactic treatment with daptomycin in 25 out of 27 patients who were scheduled to undergo liver transplant and who were VRE positive. Among those who received daptomycin, no infections related to VRE, or death, were seen within 90 days of liver transplant. The two patients who did not receive daptomycin developed VRE bacteremia early post liver transplant [61]. This is not to say that every patient who enters with risks for VRE or MDRE should receive treatment with daptomycin. Antibiotics should always be used carefully and strategically to avoid the selection for drug-specific, non-susceptible bacterial isolates such as daptomycin-resistant enterococcus. For this reason, it is important to identify patients based on their comorbidities and risk factors, including previous antibiotic use, amount of time spent hospitalized, admission to ICU, indwelling catheters, chemotherapy, or transplants. In these cases, fecal samples and rectal swabs should be collected to screen patients for genes unique to VRE (i.e., vanA, vanB). Early testing can expedite the use of appropriate treatment and reduce the rate of SSI associated with MDRE in the hospital setting.

## 10. Prevention

The source of enterococcal infection in patients is associated with the patient’s microbiota itself, more specifically the microbiota of the oral cavity, skin, intestinal contents, urethra, genitals, rectum, and perineum. Complicated infections with *Enterococcus*, specially VRE, have been found to be more common in specific populations, such as in immunocompromised patients [62]. Even if the bacteria are endogenous, the hospital environment and the severity of patient illness may render these endogenous commensals more invasive. Surgical site infections most commonly have a polymicrobial origin and contain organisms such as *Staphylococcus aureus, Escherichia coli, Pseudomonas aeruginosa,* and *Klebsiella*, some of which are consistently present on a patient’s skin. Any chemical agent for microbial reduction of the skin ideally kills all skin organisms, is nontoxic and hypoallergenic, does not result in significant systemic resorption, has residual activity, is safe for repetitive use, and can be recommended before any major or minor surgery to reduce chances of postoperative surgical site infection [2].

The large increase in gentamicin-resistant enterococcal SSIs was due to transmission by direct contact with medical personnel [10]. An increased risk of SSI with enterococcal variants, including VRE, has been also found in patients with predisposing conditions such as obesity, malnutrition, hyperglycemia, and chronic diseases. 

In the past, healthcare workers and surgical instruments have also been found to be important carriers of enterococcal infections when precautionary measures are not adequately implemented to prevent transmission of these pathogens from one patient to the next. Even more paradoxical is the fact that healthcare workers also have increased propensity for contracting infection with MDRE. Perhaps the identification of VRE-positive patients can aid the expenditure of resources in a wise manner [63]. In preparation for surgery, it is advisable to avoid shaving patients as this can potentially cause abrasions that break the skin barrier and increase susceptibility to infection. Instead, avoid removing hair at all costs, but, if necessary, a trimmer can be used to clear hair away [2]. When preparing the patient’s skin, preparations of chlorhexidine or iodine with alcohol versus chlorhexidine or iodine alone seem to work better; however, the effects of these chemicals vary depending on concentration, temperature, level of acidity, and contact time [2].

*Enterococcus*, in addition to Gram-negative and skin microbes, is more likely to be a source of SSIs in clean-contaminated procedures [60]. This results from intentional, controlled entry into a hollow viscus (respiratory, alimentary, genital, or urinary tract) without subsequent contamination [2]. Therefore, the procedures most commonly associated with *Enterococcus* are gastroduodenal procedures (bowel resections, revision surgeries for stricture repair, percutaneous endoscopic gastrectomy (PEG) insertion, pancreatoduodenectomy, bariatric procedures), small bowel procedures (incision, resections of small intestine, including enterectomies, intestinal bypasses, strictureoplasty), except small-to-large bowel anastomoses, appendectomies, hernia repairs, cesarean deliveries, hysterectomies, urologic procedures, lung, and heart–lung transplantations, liver transplantations, and kidney transplants. Anecdotally, colorectal procedures are generally not associated with enterococcal infection and, instead, are associated with anaerobes [60].

Carefully picked antibiotic prophylaxis and measures to interrupt indirect contact transmission are the main components for the control and prevention of VRE and MDRE. Acquisition of enterococcal infection has also been associated with patients who have received long courses of antibiotics in the past [28]. A longitudinal study in which 94 patients undergoing allogenic hematopoietic stem cell transplantation found an association between intestinal microbiota domination by *Enterococcus* and reduced survival. It was found that the intestinal microbiome is largely changed using antibiotics such as metronidazole and vancomycin, which not only confers an advantage to *Enterococcus* against anaerobic bacteria, but also increases the risk of acquisition of VRE [64].

## 11. Conclusions

Enterococcus is a resilient organism in the hospital environment. The increased appearance of multidrug-resistant, Gram-positive bacteria such as *Enterococcus* has compromised the healthcare system’s ability to care for patients even more. *E. faecalis* and *E. faecium* are most notable as they are widely regarded as the most common species from the Enterococcus genus causing disease. Correct identification of bacterial species within the genus *Enterococcus* is important as it may affect the use of appropriate antibiotic therapy. Enterococci are significant human pathogens which increasingly becoming resistant to multiple antimicrobials that patients receive after undergoing surgeries [23]. SSIs account for 15% of all nosocomial infections, which has led to an unexpected rise in expenses, use of more resources, and increased morbidity and mortality of patients [3,4,5]. 

The intrinsic resistance of enterococci to various antibiotics, and their ability to alter their genome, and widespread use of antibiotics in both human and veterinary medicine are the main factors driving the emergence of MDR nosocomial enterococcal infections [65]. Prevention, and the appropriate antibiotic treatment selection, could help reduce the rate of SSI associated with MDRE in the hospital setting. Given that MDRE infections significantly increase hospital costs, hospital personnel should implement control measures to prevent MDRE transmission.

Enterococci will remain an important nosocomial pathogen for the future. Continued study of enterococcal MDR mechanisms and effective treatment options is needed to increase our understanding of bacterial evolution and its complex resistance patterns.

## Figures and Tables

**Table 1:** *Enterococcus* in surgical infections.

Year	Author	Study Type	Primary Outcome/Goal
1995	J. E. Patterson	prospective, observational study (n = 110)	Antibiotic resistances among the infections included gentamicin (26%), ampicillin (10%), and vancomycin (8%). Clinical cure was achieved in 64% with overall mortality of 23%. Ampicillin resistance was highly predictive of lack of cure.
1997	M. E. Klepser	randomized clinical trial (n = 10)	Use of shorter dosing intervals of piperacillin–tazobactam oricarcillin–clavulanate should be considered in combination with an aminoglycoside to improve the bactericidal profiles of these agents for E. faecalis.
2002	A. Sitges-Serra	A prospective longitudinal observational (n = 200)	Subjects with enterococcal SSI infections have a higher incidence of multiple infections, and the majority develop at least one polymicrobial infection at the surgical site, and postoperative enterococcal infections were associated with a high mortality rate (21% vs. 4%; *p* < 0.0007).
2003	S. S. Min	case report (n = 1)	Multidrug-resistant E. faecium strains demonstrate resistance to linezolid, and quinupristin/dalfopristin may emerge during therapy with these agents, further limiting therapeutic options.
2008	V. Savini	case report (n = 1)	Daptomycin can be a promising alternative in therapy of severe, difficult-to-treat enterococcal infections.
2016	W. Dessie	cross-sectional study (n = 107)	The practice of aseptic technique during and after surgery should be the primary support rather than overreliance on antibiotics to reduce emergence and spread of resistant pathogens.
2017	J. Pochhammer	retrospective chart review (n = 2713)	Perioperative antibiotic prophylaxis by the additional administration of ampicillin or vancomycin could be advantageous.
2017	M. Tamura	case report (n = 1)	Prophylactic broad-spectrum antibiotics used for c- sections could lead to postpartum nosocomial enterococcal bacteremia.
2018	R. A. Heitkamp	prospective, longitudinal, observational (n = 200)	Approximately 60% of case subjects had three or more infections, and 91% had one or more polymicrobial infection. Frequent co-colonizing microbes in polymicrobial wound infections with Enterococcus were other ESKAPE pathogens (64%) and fungi (35%).
2019	W. Albishi	cross-sectional study (n = 119)	Level of knowledge about SSIs and risks of wound infections among medical physicians should be improved to ensure better wound care and quality care for the patients.

**Table 2:** Multidrug-resistant pathways in *Enterococcus*.

Site of Resistance	Strategy	Antibiotic	Genes or Gene Products
Cell Wall	Decreased affinity for PBPs	β-lactams	pbp5
Cell Wall	Drug inactivation	β-lactams	blaZ
Cell Wall	Cell signaling	Cephalosporins	croRS and ireK
Cell Wall	Altered target	Glycopeptides	van operons
Ribosome	Decreased drug uptake	Aminopenicillins	-

## Data Availability

Not applicable.
